# Supplementary material for: 14-3-3 proteins activate Pseudomonas exotoxins-S and -T by chaperoning a hydrophobic surface
Source: Nat Commun. 2018 Sep 17;9:3785. doi: 10.1038/s41467-018-06194-1 (PMC6141617; doi:10.1038/s41467-018-06194-1)
Supplement: Supplementary file 1 — Supplementary Information [file 41467_2018_6194_MOESM1_ESM.pdf]

Supplementary Information accompanying

14-3-3 proteins activate *Pseudomonas* exotoxins-*S* and –T by chaperoning a hydrophobic surface

Karlberg et al.

Table of Contents

Figure 1 – Apparent affinities of ExoS for seven 14-3-3 isoforms 3
Table 1 – Data collection for initial ExoS:14-3-3β complex crystal 4
Figure 2 – Interactions between ExoS and ligands in the active site 5
Figure 3 – Higher molecular weight exotoxin:14-3-3β complexes 6
Figure 4 – Purity analysis of ExoS:14-3-3β complex column fractions 7
Figure 5 – Stereo images of the aligned structures of ExoS and ExoT 8
Figure 6 – Sequence alignments of 14-3-3 dependent exotoxins 9
Figure 7 – Putative protein-protein interaction inhibitors examined 10
Figure 8 – Fluorescence anisotropy measurements of ExoS binding to 14-3-3 11
Figure 9 – Rationale for design of ExoS NAD binding site mutants 12
Table 2 – Kinetic constants for ExoS NAD binding site mutants 13
Figure 10 – ADP-ribosylation of K-Ras by the ExoS ART domain 14
Figure 11 – ADP-ribosylation of K-Ras by the C-terminal deletion construct, $ExoS^{233\text{-}435}$ 15
Figure 12 – ADP-ribosylation of K-Ras by the C-terminal deletion construct, $ExoS^{233\text{-}419}$ 16
Figure 13 – ADP-ribosylation of Rac3 by the ExoS ART domain 17
Figure 14 – Heat induced aggregation of ExoS 18
Table 3 – Heat induced aggregation of ExoS 19
Figure 15 – Comparison of 14-3-3β:ExoS with four 14-3-3 client complexes 20
Figure 16 – Revised positioning of the substrate binding region in ExoS and –T 21
Table 4 – Oligonucleotide primer sequences 22
Table 5 – Yeast strains generated 22
Supplementary Methods:
Crystallization of ExoS:14-3-3β and ExoT:14-3-3β complexes 23
Carba-NAD synthesis 24
Figure 17 – Synthesis of carba-NAD 28
Figure 18 – NMR spectra for carba-NAD 29
Supplementary references 30

a

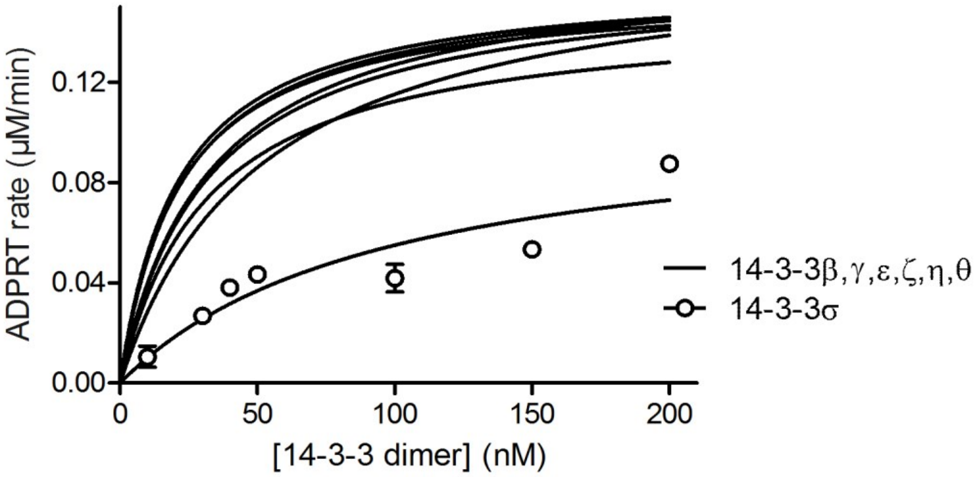

b

**14-3-3β**	**14-3-3θ**	**14-3-3γ**	**14-3-3ε**	**14-3-3η**	**14-3-3σ**	**14-3-3ζ**
**22.6±3.2;**<br>**n=4;**<br>**R^2=0.940**	34.7±8.5;<br>n=2;<br>R^2=0.900	34.4±6.9;<br>n=2;<br>R^2=0.936	47.1±10.2;<br>n=2;<br>R^2=0.937	35.9±5.4;<br>n=2;<br>R^2=0.962	109.6±9.9;<br>n=2;<br>R^2=0.811	33.5±3.9;<br>n=4;<br>R^2=0.989

Supplementary Figure 1

Ability of seven human 14-3-3 isoforms to stimulate ExoS glycohydrolase activity. (**a**) Etheno-NAD glycohydrolase activity of 50 nM $ExoS^{233\text{-}453}$ in presence of 7 human 14-3-3 isoforms at various concentrations. For clarity, only best-fit trendlines of the data are shown for 6 of the proteins (see **Figure 3e** of the main text for details on 14-3-3β and 14-3-3ζ binding). (**b**) Apparent affinities for human 14-3-3 isoforms (K_{app}, in nM), estimated from the data shown in panel a. Assay rate data were fitted to a one-site specific binding model. Means ± standard errors are reported.

Supplementary Table 1: Data collection for initial ExoS:14-3-3β complex crystal[a]

**Data collection**	**ExoS E379A,E381A apo:14-3-3β heterotrimer**
beam line	Bessy, BL14.1
wavelength (Å)	0.91841
space group	C2
unit cell dimensions (Å,Å,Å,°,°,°)	134.46, 57.31, 128.67, 90, 112.02, 90
resolution (Å)	48.31-3.22 (3.41-3.22)
unique reflections	14841 (2285)
R(merge)	0.027 (1.39)
completeness (%)	98.5 (95.9)
redundancy	6.7 (6.6)
<I>/<σI>	7.9 (1.4)
CC(1/2)	0.997 (0.652)

[a]Data collection statistics for the molecular replacement template for the structures reported in Table 1 of the main text. Data in parentheses refer to the highest resolution shell.

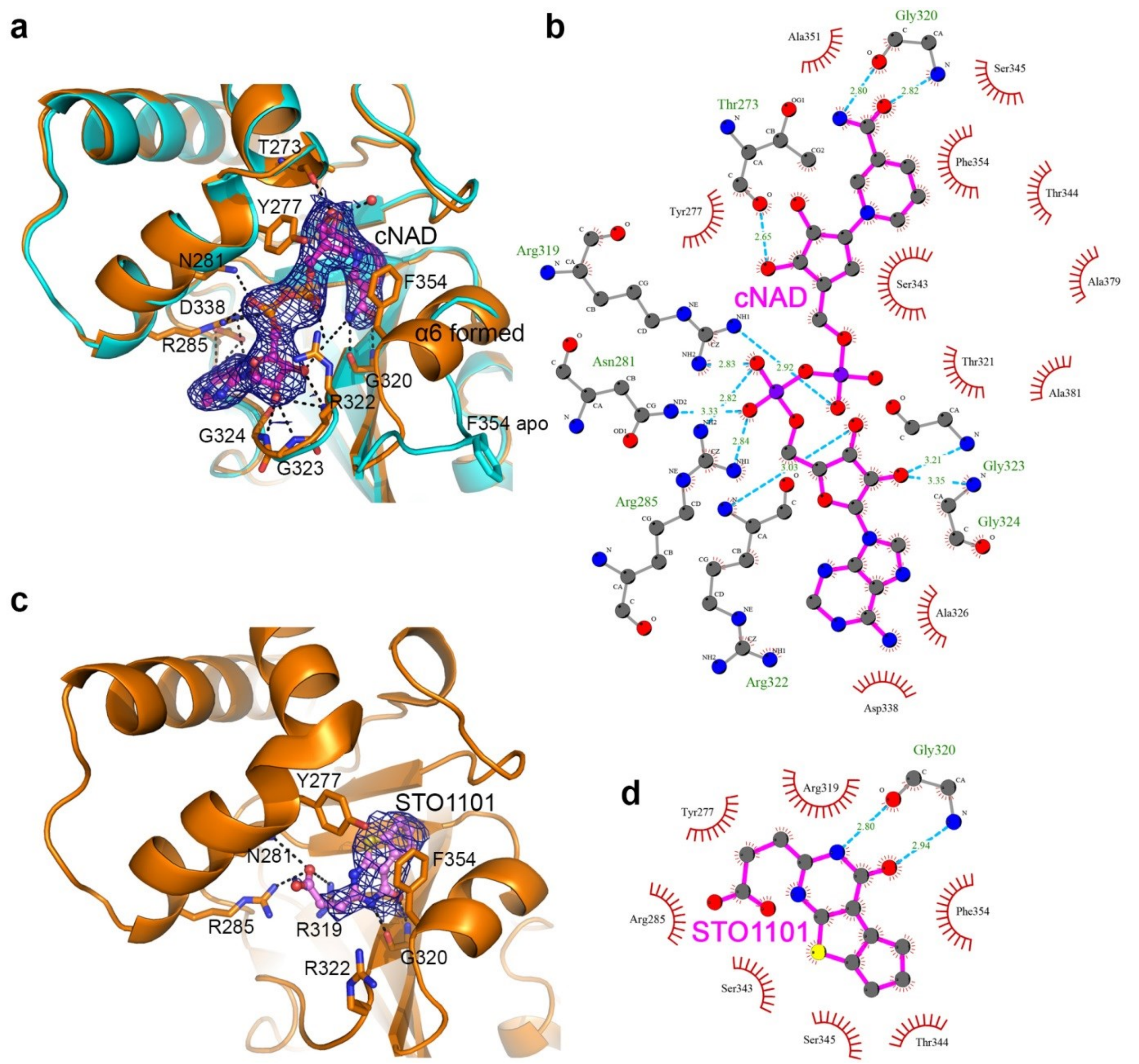

Supplementary Figure 2

Interactions between ExoS and ligands in the active site. Note that the side chains of E379 and E381 were mutated to alanines in order to obtain recombinant protein for crystallization. (**a**) Comparison between the *apo* (cyan) and carba-NAD bound (gold) structures of ExoS. A simulated annealing 2F_{obs}-F_{calc} composite omit electron density map contoured at 1.2σ around carba-NAD is shown. F354 side chain engagement in ligand binding is required to form α-helix 6. (**b**) 2D representation of ExoS:carba-NAD interactions. (**c**) Structure of ExoS with bound inhibitor STO1101. A simulated annealing 2F_{obs}-F_{calc} composite omit electron density map contoured at 1.2σ around STO1101 is shown. (**d**) 2D representation of ExoS:STO1101 interactions. Note that STO1101 is a previously characterized inhibitor of ExoS and ExoT with potency in the low micromolar range.[1, 2] Panels b and d were prepared using LigPlot+.[3]

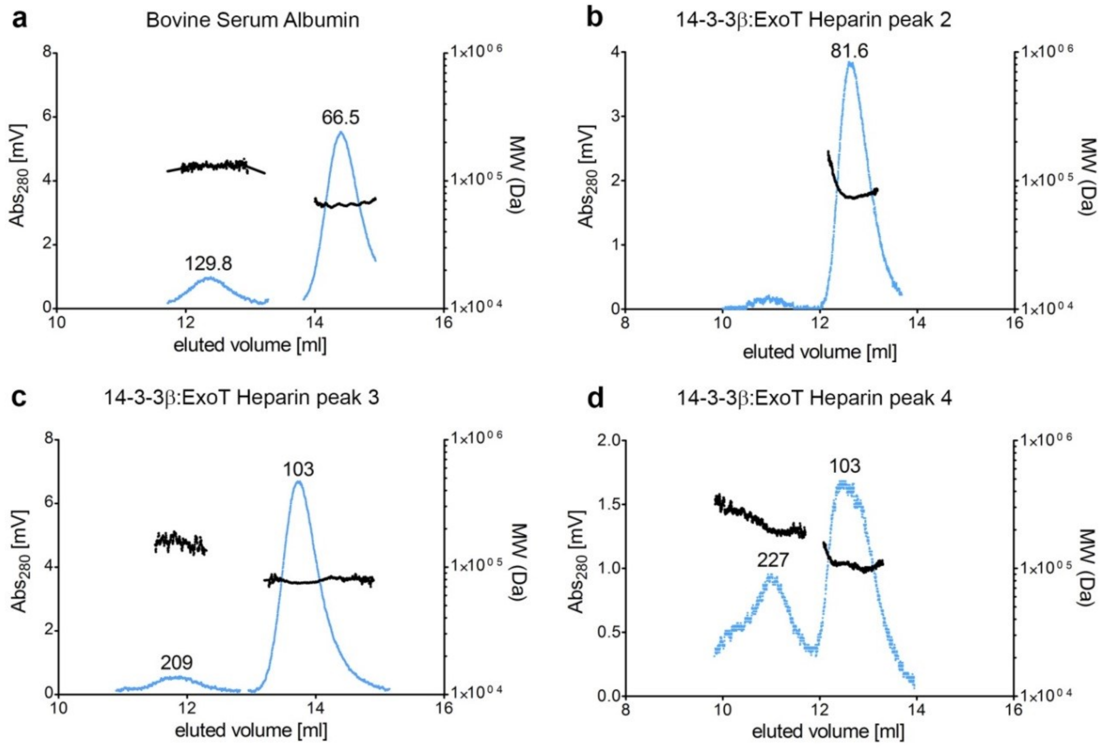

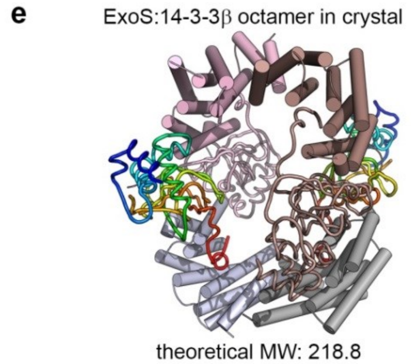

f

Size	Formula	Stable	Surface area, Å²	Buried area, Å²	ΔG_{int}, kcal/mol	ΔG_{diss}, kcal/mol
8	A_4B_4	Yes	72680	22770	-76.7	6.8
4	A_2B_2	Yes	39370	8340	-35.7	0.8
4	A_2B_2	Yes	39230	8520	-36.3	0.8

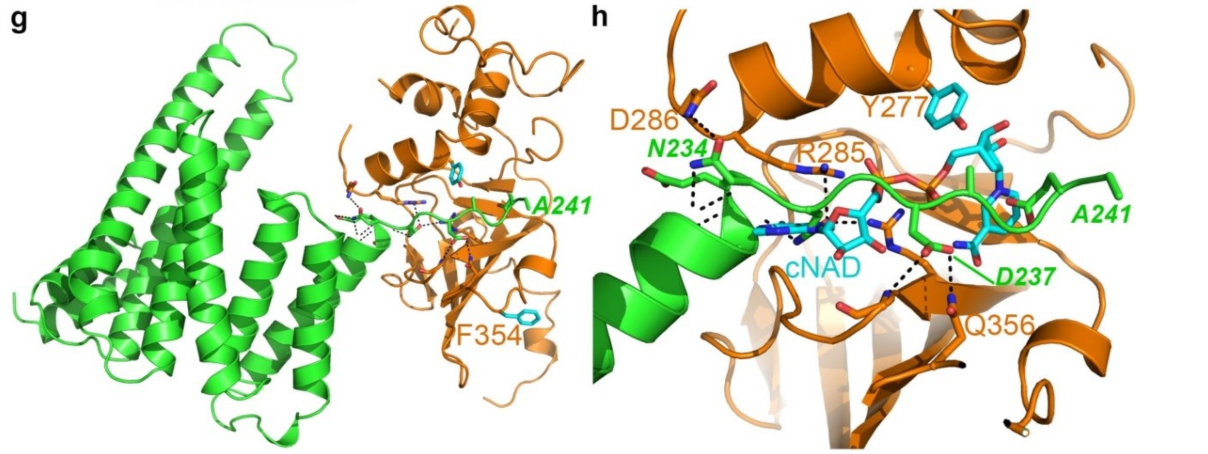

Supplementary Figure 3

Size distribution of exotoxin complexes. (**a**) SEC-RALS control experiment showing monomeric and dimeric species of BSA and their molecular weights calculated using OmniSEC software (Malvern Instruments). (**b-d**) Size distribution of ExoT:14-3-3β complex protein from three consecutive heparin chromatography peaks. (**b**) The heterotetrameric

ExoT:14-3-3β complex that resulted in the crystal structure PDB entry 6GNN (depicted in **Fig. 1h** of the main text). (**c**) Peak 3 consisting of complexes consistent in size with heterotetramers and heterooctamers. (**d**) Peak 3 containing a larger fraction of complexes consistent in size with heterooctamers. (**e**) In the crystals of the heterotetrameric ExoS:14-3-3β complex, crystal contacts show formation of heterooctamers. (**f**) Interface analysis of the crystallographic heterotetramers and heterooctamers using the PISA server[4] suggests these assemblies may be stable in solution. (**g**) Initially, complex crystals were obtained with 14-3-3β containing the full native C-terminus. In these crystals, the active site of ExoS (gold) was occupied by the C-terminus of 14-3-3β from a neighbor unit cell (green). This observation might provide another mechanism for the formation of larger molecular weight complexes in solution. This initial structure formed the basis for our decision to attempt complex crystallization using a shorter 14-3-3β construct (residues 1-234). Incidentally, the two C-terminal residues of the initial 14-3-3β constructs (E240, A241) are a cloning artifact. (**h**) Detail of the active site occupied by the 14-3-3β C-terminus. For clarity, the position of Carba-NAD is shown.

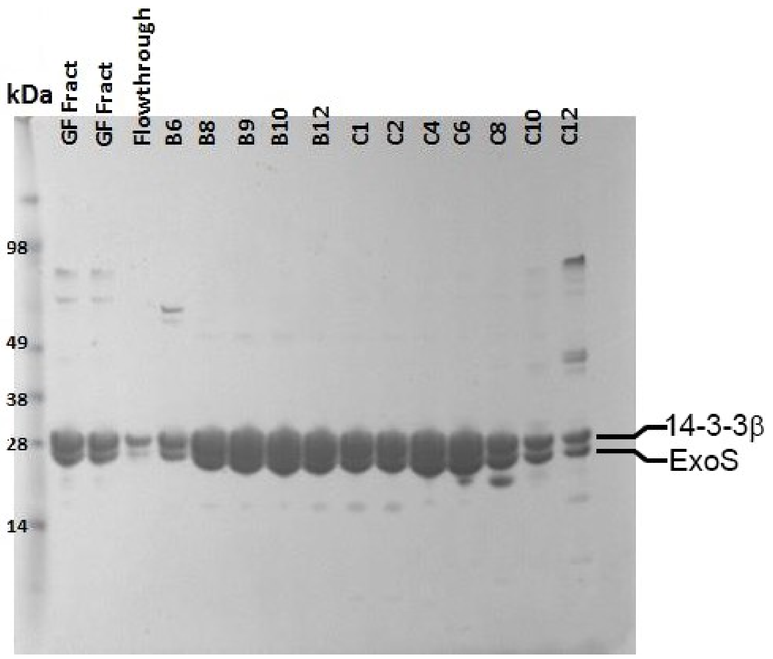

Supplementary Figure 4

Coomassie stained SDS-PAGE gel showing an analysis of ExoS:14-3-3β complex column fractions. Protein complex was isolated from *E.coli* lysates by IMAC, and subjected to SEC, as described in the methods section. The figure shows the purity of the material that was loaded onto a heparin column ("GF fract") and of the material that passed through the heparin column ("Flowthrough"). Lanes B6 thru C12 show samples of the fractions that were eluted when the heparin column was developed with a linear salt gradient. The material peaking in fraction B10 was heterotrimeric complex, and the material peaking in fraction C4 was heterotetrameric complex, shown by both SEC-RALS and X-ray crystallography. The complex runs similar as the 28 kDa size marker, where the upper band represents 14-3-3β and the lower band represents hexahistidine-tagged ExoS$^{233\text{-}453}$ (indicated).

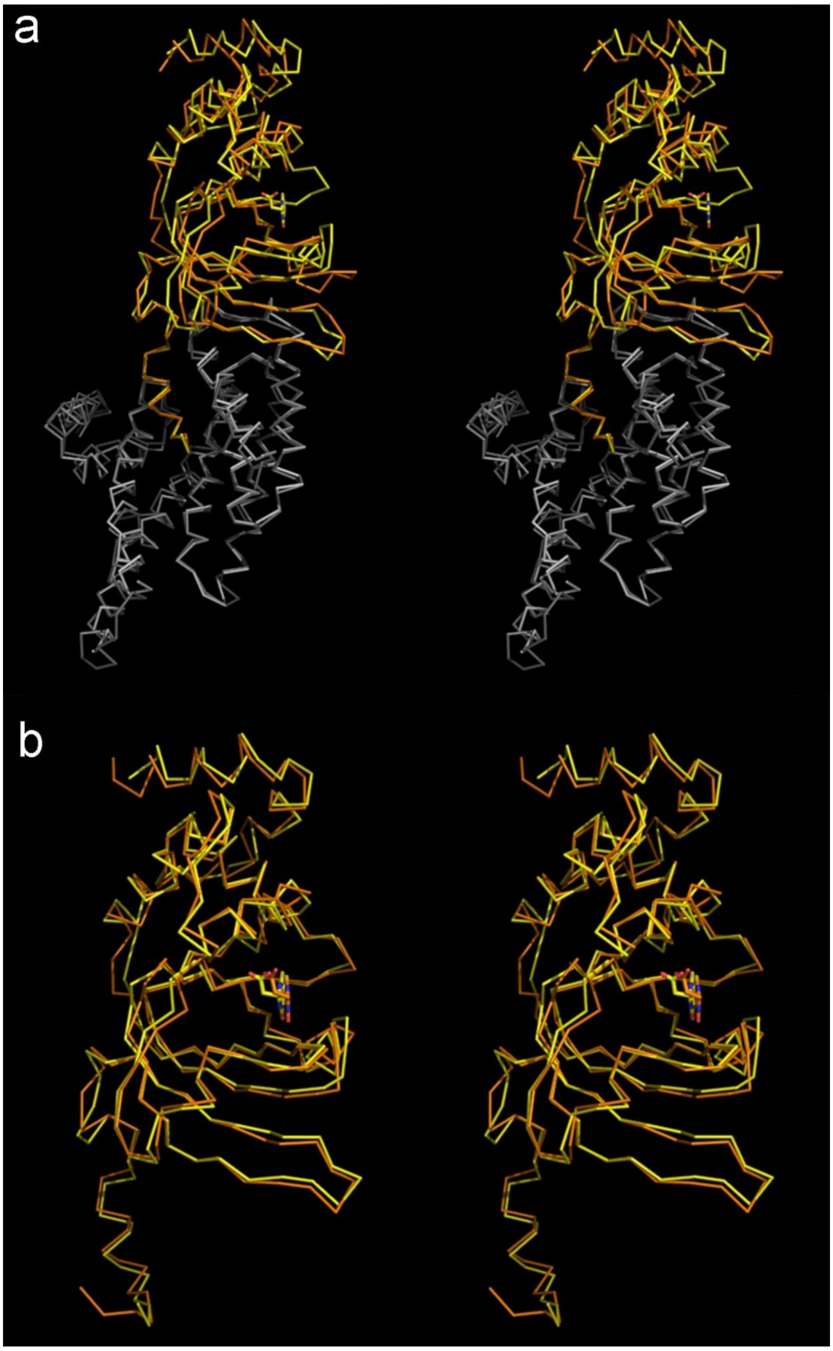

Supplementary Figure 5

Stereo images of the aligned structures of ExoS and ExoT. (**a**) Superposition of the 14-3-3β:exotoxin heterodimers from the tetrameric complexes (PDB: 6GN0 and 6GNN). ExoS (orange; 14-3-3β in dark grey) secondary structural elements do not arrange around the empty active site (as compared to the occupied site in ExoT), resulting in a relatively high overall RMSD of 1.84Å. ExoT is shown in yellow, ExoT-bound 14-3-3β in light gray. (**b**) The ART domains of ExoS and ExoT in complex with STO1101 (PDB: 6GNJ, 6GNN) align with an RMSD of 0.97 Å. The inhibitor is shown as sticks.

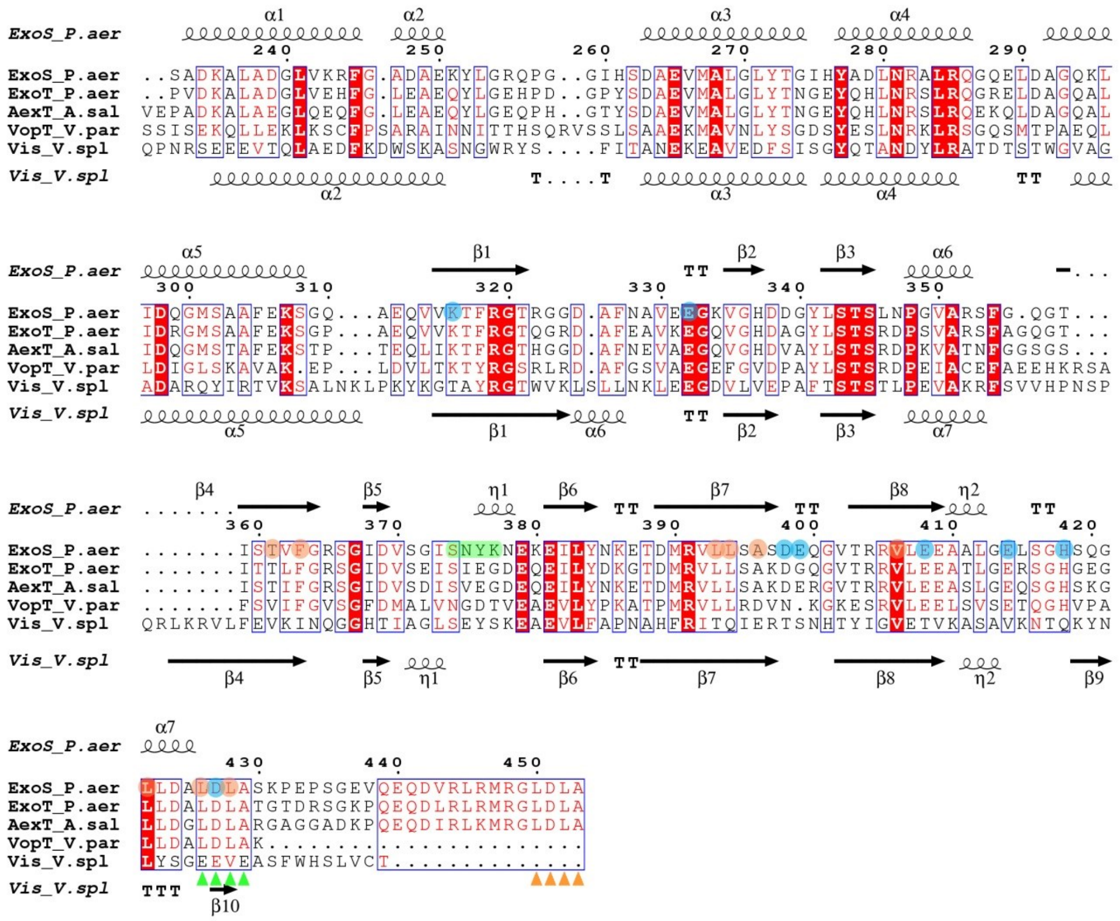

Supplementary Figure 6

Sequence alignments of 14-3-3 dependent exotoxins. A multiple alignment of the ART domains of four 14-3-3 dependent toxins (*Pseudomonas aeruginosa* ExoS and ExoT; *Aeromonas salmonicida* AexT; *Vibrio parahaemolyticus* VopT) and one four 14-3-3 independent toxin (*Vibrio splendidus* Vis) was prepared using Clustal Omega[5] and the secondary structural elements for ExoS and Vis (this study and Merrill and co-workers,[6] respectively) were added using ESPript.[7] The positions of LDLA-boxes 1 and -2 are indicated by green and orange arrowheads, respectively. ExoS sidechains that interact with 14-3-3β in our crystal structures are highlighted (orange, hydrophobic interactions; blue, ionic interactions). Note the conservation of residues at these positions among ExoS, ExoT, and AexT; the partial conservation in VopT; and the general lack of conservation in Vis. The ARTT loop is highlighted in green.

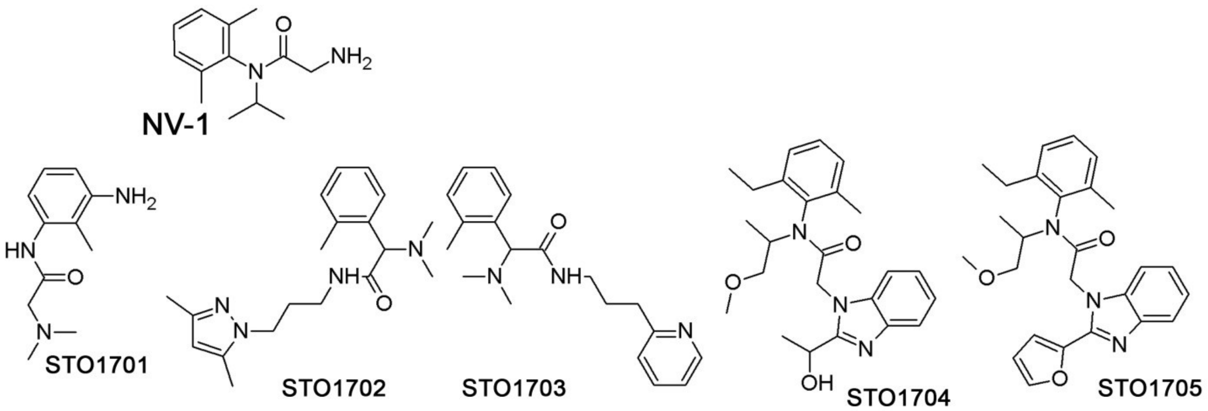

Name	Source	Source ID	Cpd Name	Smiles	ExoS IC50 (μM)| pIC50 (M)	ExoT IC50 (μM)| pIC50 (M)
**NV-1**	Enamine	FCH3171585	2-amino-N-(2,6-dimethylphenyl)-N-(propan-2-yl)acetamide	CC(C)N(C(=O)CN)c1c(C)cccc1C	n.a.	n.a.
**STO1701**	Hit2Lead	4027798	N~1~-(3-amino-2-methylphenyl)-N~2~,N~2~-dimethylglycinamide	C(C)1=C(C=CC=C1NC(CN(C)C)=O)N	n.b.	n.d.
**STO1702**	Hit2Lead	48166093	2-(dimethylamino)-N-[3-(3,5-dimethyl-1H-pyrazol-1-yl)propyl]-2-(2-methylphenyl)acetamide	N1=C(C)C=C(C)N1CCCNC(=O)C(N(C)C)C1C=CC=CC=1C	n.b.	n.d.
**STO1703**	Hit2Lead	88817229	2-(dimethylamino)-2-(2-methylphenyl)-N-(3-pyridin-2-ylpropyl)acetamide	C(N(C)C)(C1C=CC=CC=1C)C(NCCCC1C=CC=CN=1)=O	n.b.	n.d.
**STO1704**	Hit2Lead	6944250	N-(2-ethyl-6-methylphenyl)-2-[2-(1-hydroxyethyl)-1H-benzimidazol-1-yl]-N-(2-methoxy-1-methylethyl)acetamide	N1(C2=CC=CC=C2N=C1C(C)O)CC(=O)N(C(C)COC)C1=C(CC)C=CC=C1C	638 | 3.197±0.066 R^2 = 0.859	726 | 3.139±0.101 R^2 = 0.843
**STO1705**	Hit2Lead	7725255	N-(2-ethyl-6-methylphenyl)-2-[2-(2-furyl)-1H-benzimidazol-1-yl]-N-(2-methoxy-1-methylethyl)acetamide	N1(C2=CC=CC=C2N=C1C1=CC=CO1)CC(=O)N(C(C)COC)C1=C(CC)C=CC=C1C	n.b.	n.d.

Supplementary Figure 7

Putative protein-protein interaction inhibitors examined for effects on the 14-3-3β stimulated activity of ExoS. Top, chemical structures of PPI inhibitor NV-1[8] and five related compounds. Table, identities and sources of the compounds tested, and outcome. Abbreviations: n.a., compound not available; n.b., no inhibition detected (up to a compound concentration of 1 mM in presence of 3% DMSO); n.d., not determined.

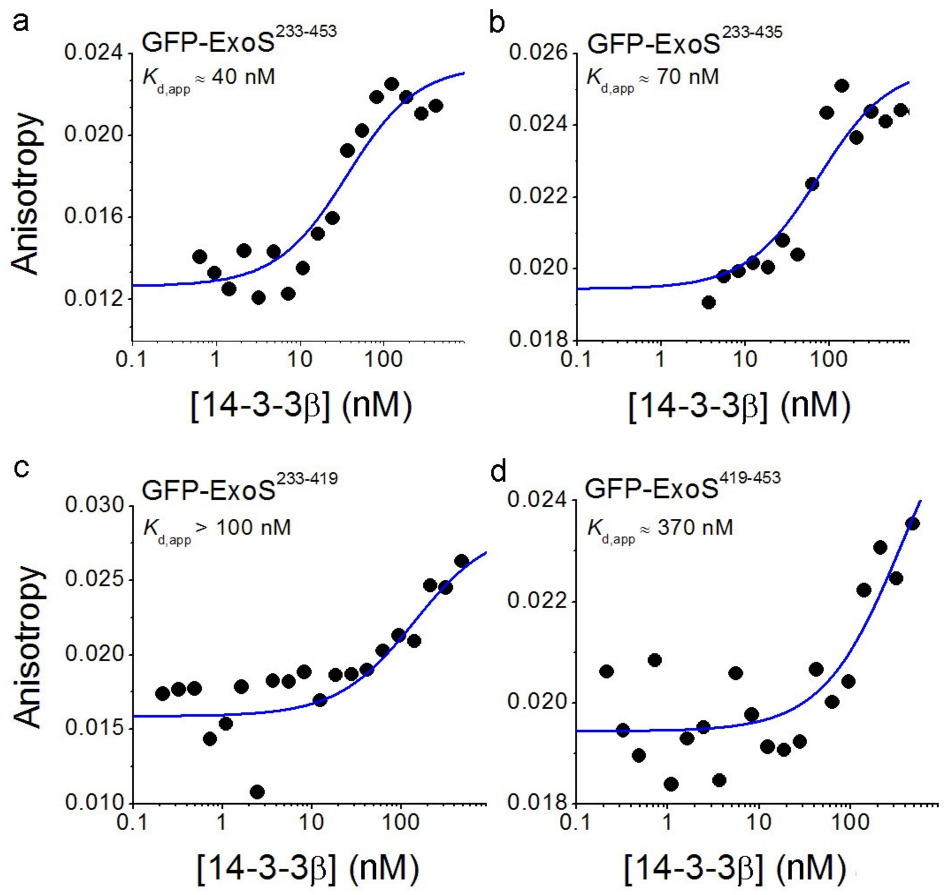

Supplementary Figure 8

Binding of GFP-tagged ExoS C-terminal truncation constructs to 14-3-3β. N-terminal green fluorescent protein fusions of the ExoS ART domain (a) or ART domain lacking the amphipathic LDLA-box 2 (b) or LDLA-boxes 1 and 2 (c) were titrated with 14-3-3β protein and fluorescence anisotropy was assessed. Note that ExoS ART domain containing LDLA-box 1 binds 14-3-3β with an apparent affinity in the mid-nanomolar range. When only the hydrophobic interaction site is present ($ExoS^{233-419}$; panel c) the apparent affinity is sub-micromolar. (d) The C-terminal peptide 419-453 of ExoS, containing both LDLA boxes, binds 14-3-3β with an apparent affinity in the sub-micromolar range. A similar result (Kd,app = 0.81 µM) was obtained for the fluorophore tagged peptide QGLLDALDLAS using the same method.[9] Experimental data were evaluated by fitting to a quadratic binding equation using Origin (see Methods section in the main text).

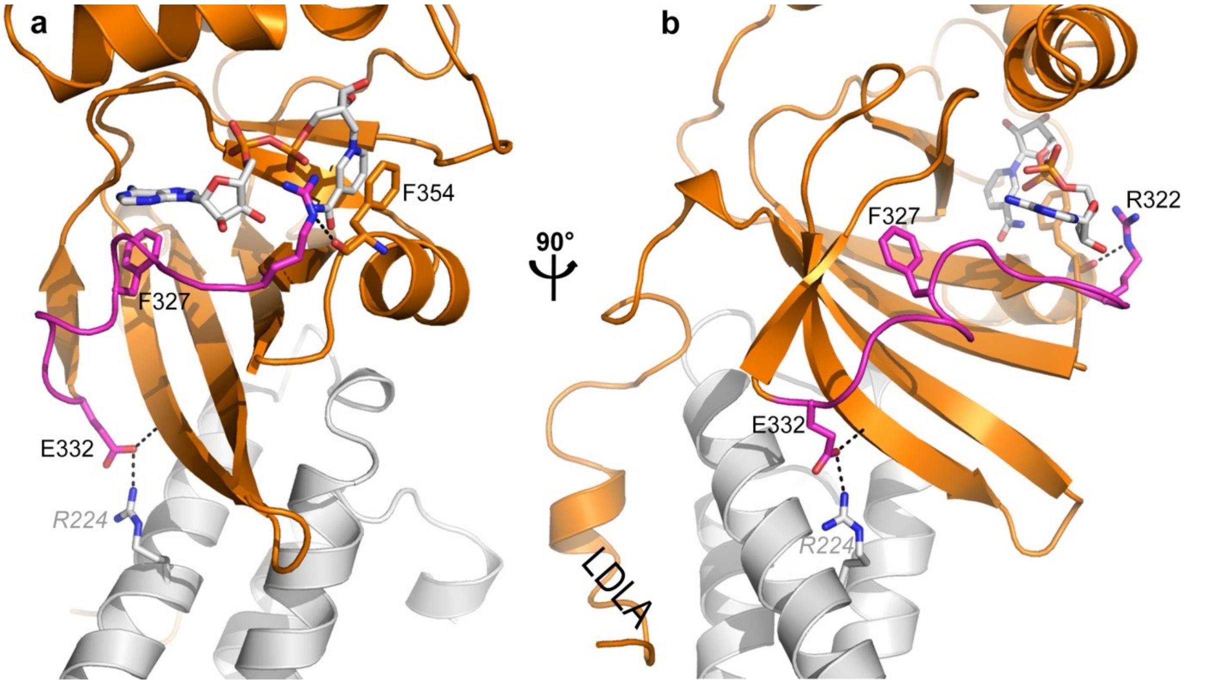

Supplementary Figure 9

Rationale for design of ExoS NAD binding site mutants. (**a**) We observed that ExoS (gold) residue E332 appeared to connect 14-3-3 to the NAD$^+$ binding site via a salt bridge with R224 of 14-3-3β (grey) and the ExoS segment shown in purple. We reasoned that 14-3-3 protein binding might activate exotoxins by allowing them to form a competent active site. To test this hypothesis, we introduced the E332A mutation in ExoS$^{233\text{-}453}$ (see **Supplementary Table 2** on the following page). (**b**) As controls for the E332A mutation, we introduced F327A and R322A. The F327 side chain links the same loop (purple) to the hydrophobic core of the domain, beneath the NAD$^+$ binding site. The R322 side chain contributes to NAD$^+$ binding by closure of the loop. We reasoned that the F354 side chain was necessary for NAD$^+$ binding, as it stacks with the nicotinamide, and as the helical fragment only folds in the presence of ligand (panel a; see also **Supplementary Fig. 2a** above). The position of LDLA box-1 is indicated in panel b for orientation.

Supplementary Table 2: Kinetic constants for ExoS NAD binding site mutants[a]

	substrate	$K_M^{\varepsilon NAD}$ (μM)	k_{cat} (min^{-1})	k_{cat}/K_M ($\mu M^{-1}sec^{-1}$)	R^2
ExoS[233-453] wild type	K-Ras	49.0±9.1	7.9±0.5	2.68×10^{-3}	0.947
	Rnd1	39.9±18.1	7.9±1.0	3.31×10^{-3}	0.987
	Agmatine	133±14	53.5±2.2	$6.7x10^{-3}$	0.979
ExoS[233-453] R322G	K-Ras	67.9±14.2	0.80±0.06	1.97×10^{-4}	0.952
	Rnd1	93.9±24.5	0.77±0.08	1.37×10^{-4}	0.930
	Agmatine	112±6.6	5.6±0.14	8.34×10^{-4}	0.997
ExoS[233-453] F327R	K-Ras	>1000	n.d.	n.d.	0.987
	Rnd1	264±54	1.7±0.20	1.08×10^{-4}	0.979
	Agmatine	132±7.9	5.8±0.16	7.34×10^{-4}	0.997
ExoS[233-453] E332G	K-Ras	45.3±4.2	7.9±0.2	2.91×10^{-3}	0.988
	Rnd1	43.6±3.8	3.8±0.11	1.44×10^{-3}	0.989
	Agmatine	54.2±9.1	43.9±2.6	1.35×10^{-2}	0.968

[a] Calculated from ADP-ribosyltransferase activities using 14-3-3β as cofactor, εNAD^+ as co-substrate, and the indicated substrates (at a concentration near their K_M) as acceptor for the modification. Rate data (n=2) were converted to concentrations of εNAD^+ by calibration with εAMP, and fitted to the Michaelis equation. Means ± standard errors are reported.

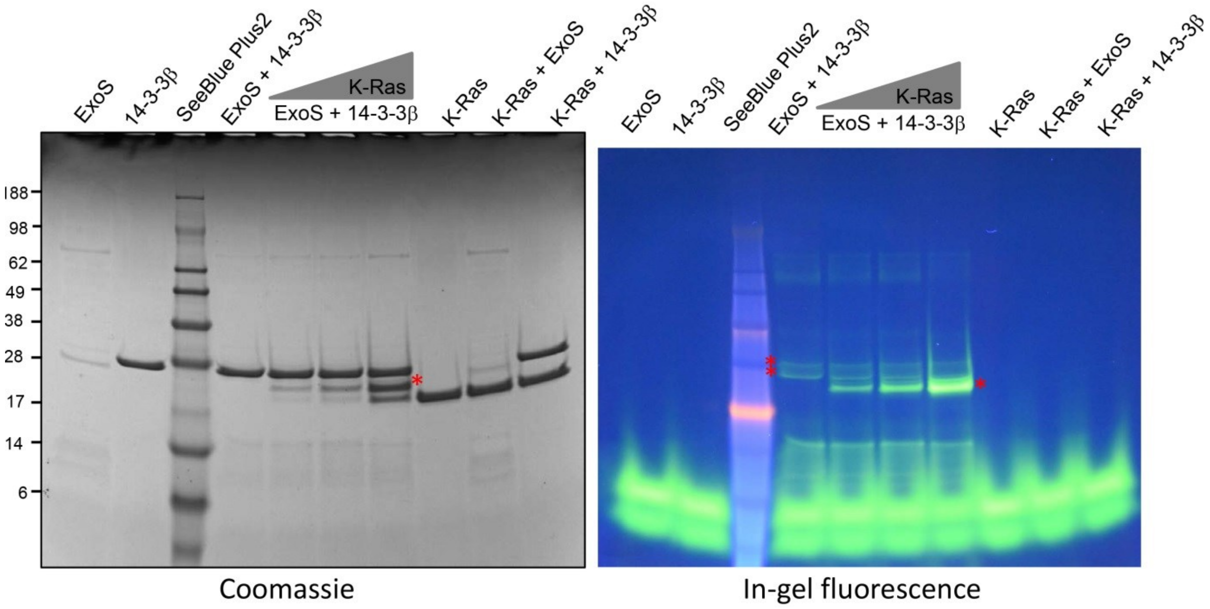

Supplementary Figure 10

ADP-ribosylation of K-Ras by the ExoS ART domain. Reactions containing (as indicated) 0.5 μM ExoS$^{233-453}$, 1.5 μM 14-3-3β, 25 μM fluo-NAD^{+}, and K-Ras (0.5, 1.0, or 5 μM) were incubated 40 minutes at room temperature. Reactions were supplemented with SDS-PAGE sample buffer, heated to 70°C for 10 minutes, and separated by SDS-PAGE on 14% Tris-glycine gels. Gels were rinsed and imaged on a UV-transluminator before being stained with Coomassie Brilliant Blue. Note the shift in the K-Ras band (indicated by a single asterisk), which is characteristic for ExoS-mediated ADP-ribosylation of K-Ras.[10] We have not been able to identify the proteins in the doublet marked by a double asterisk in the right panel.

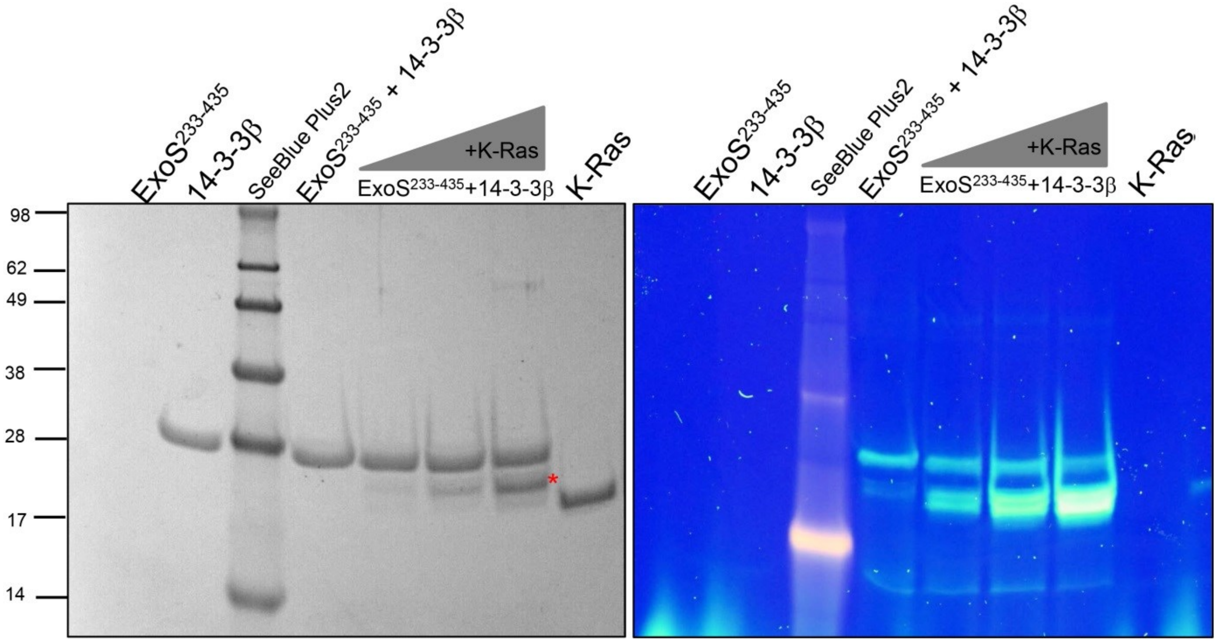

Supplementary Figure 11

ADP-ribosylation of K-Ras by the ExoS ART domain truncated at residue 435. Reactions containing (as indicated) 0.5 µM ExoS$^{233\text{-}435}$, 1.5 µM 14-3-3β, 25 µM fluo-NAD^{+}, and K-Ras (0.5, 1.0, or 5 µM) were incubated 40 minutes at room temperature. Reactions were supplemented with SDS-PAGE sample buffer, heated to 70°C for 10 minutes, and separated by SDS-PAGE on 14% Tris-glycine gels. Gels were rinsed and imaged on a UV-transluminator before being stained with Coomassie Brilliant Blue. The characteristic shift in the K-Ras band is indicated by asterisks in the left panel (cf **Supplementary Figure 10** above).

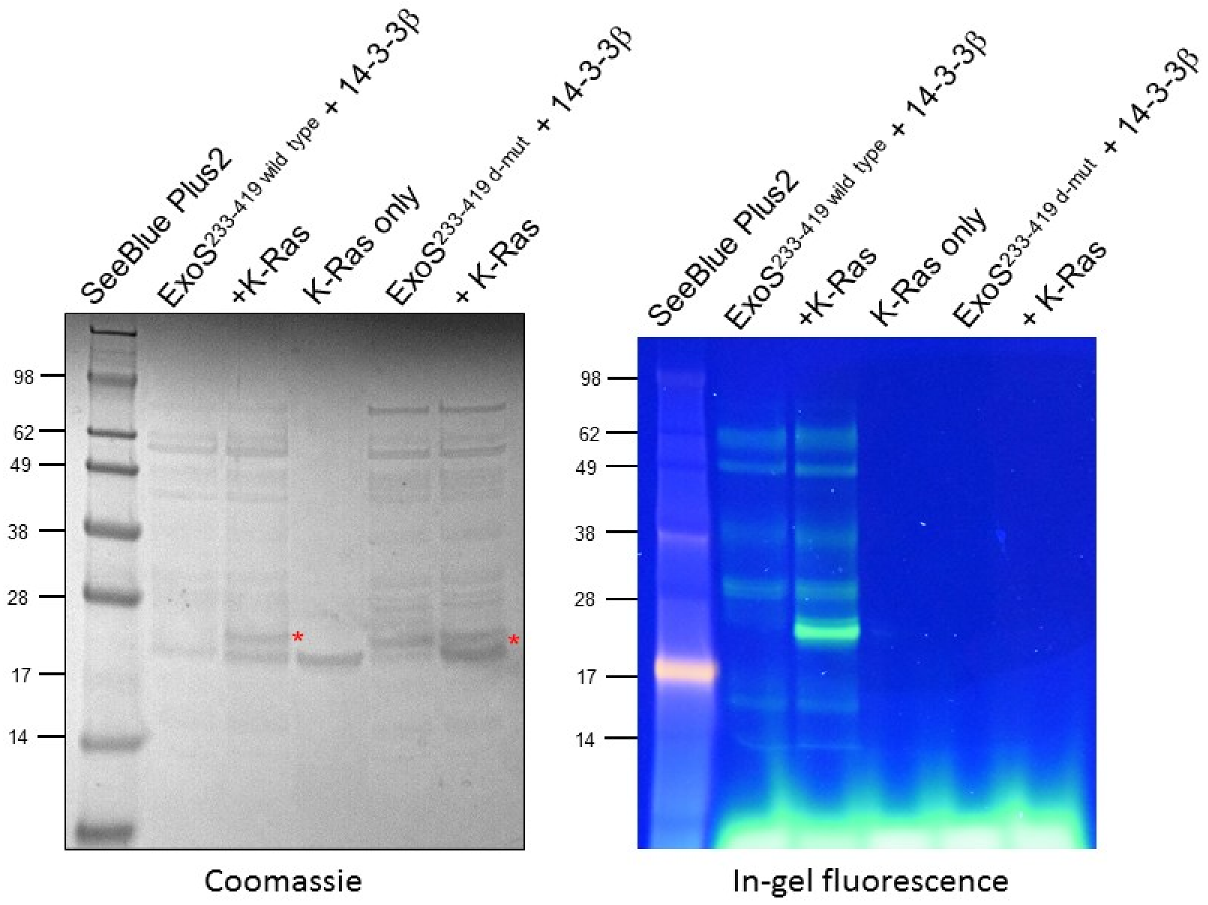

Supplementary Figure 12

ADP-ribosylation of K-Ras by the ExoS ART domain truncated at residue 419. Reactions containing (as indicated) approximately 0.5 µM ExoS$^{233\text{-}419}$:14-3-3β complex (co-expressed; either wild type or E379A,E381A double mutant), 25 µM fluo-NAD^{+}, and 1.0 µM K-Ras were incubated 40 minutes at room temperature. Reactions were supplemented with SDS-PAGE sample buffer, heated to 70°C for 10 minutes, and separated by SDS-PAGE on 14% Tris-glycine gels. Gels were rinsed and imaged on a UV-transluminator before being stained with Coomassie Brilliant Blue. The characteristic shift in the K-Ras band is indicated by asterisks in the left panel (cf **Supplementary Figure 10** above).

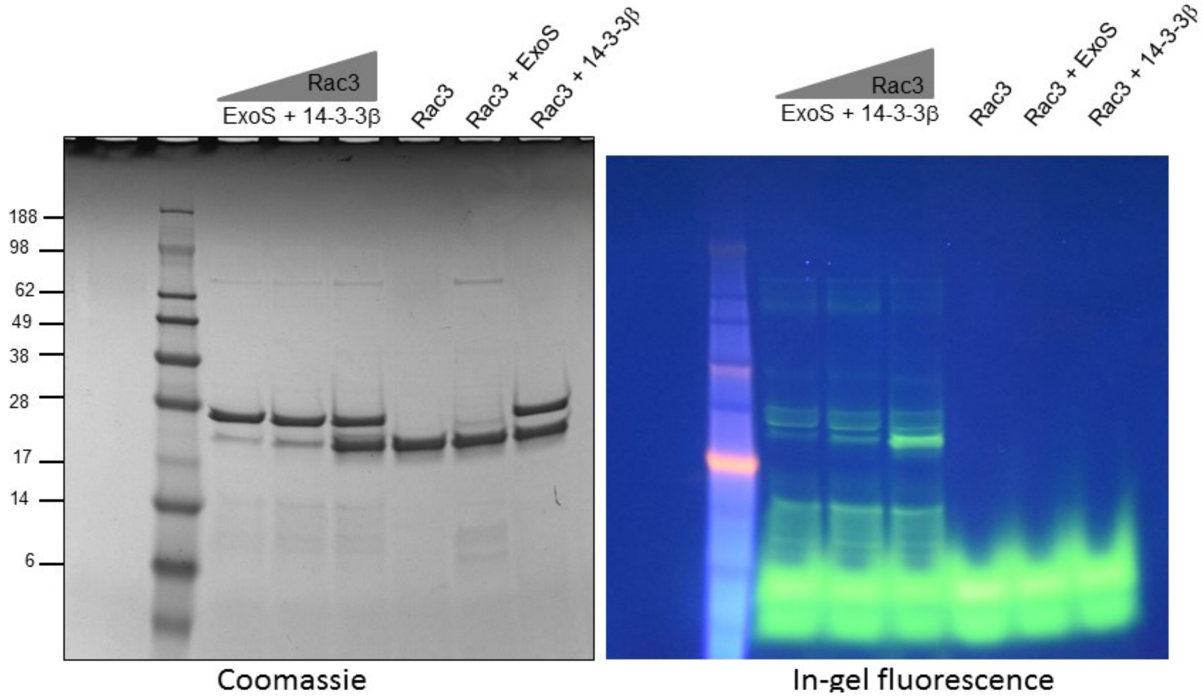

Supplementary Figure 13

ADP-ribosylation of Rac3 by the ExoS ART domain. Reactions containing (as indicated) 0.5 µM ExoS$^{233\text{-}453}$, 1.5 µM 14-3-3β, 25 µM fluo-NAD^{+}, and Rac3 (0.5, 1.0, or 5 µM) were incubated 40 minutes at room temperature. Reactions were supplemented with SDS-PAGE sample buffer, heated to 70°C for 10 minutes, and separated by SDS-PAGE on 14% Tris-glycine gels. Gels were rinsed and imaged on a UV-transluminator before being stained with Coomassie Brilliant Blue.

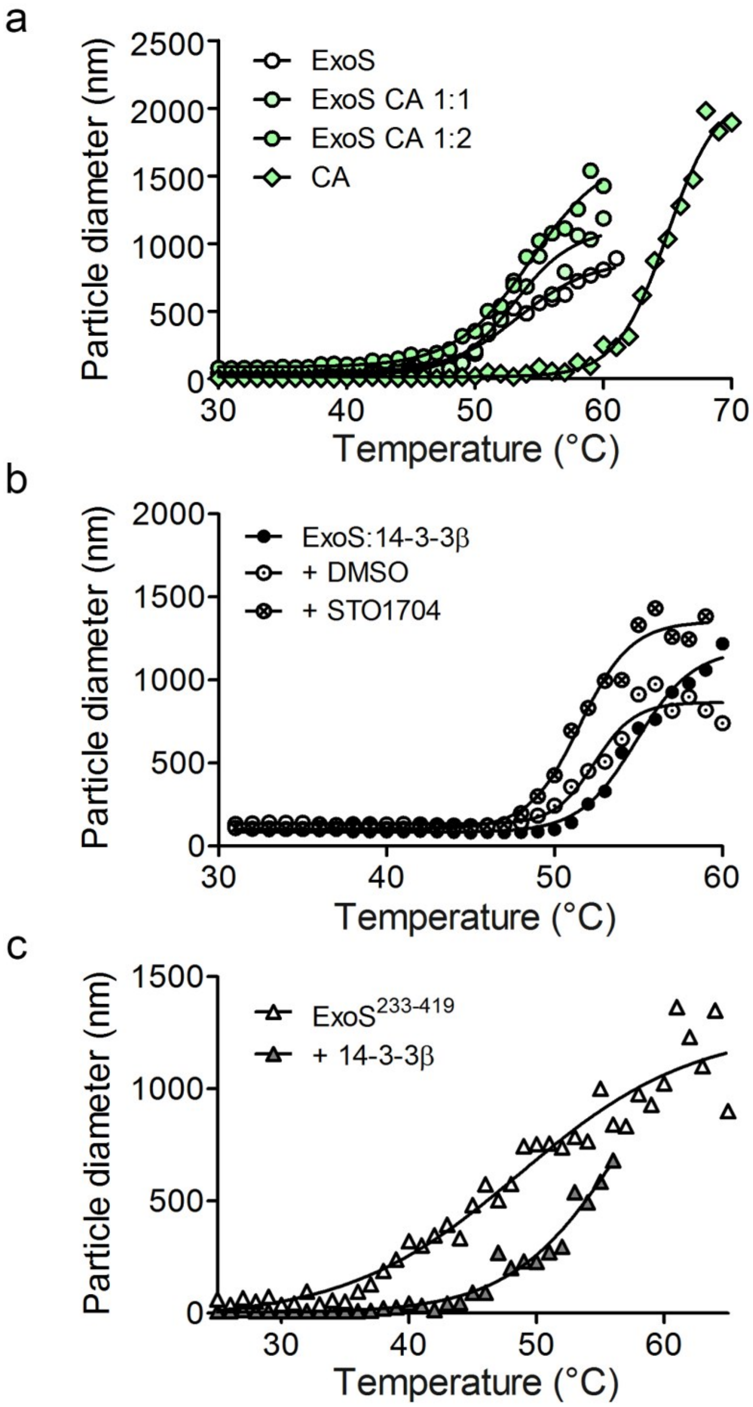

Supplementary Figure 14

Heat induced aggregation of ExoS. (**a**) Control experiment showing that carbonic anhydrase (CA), an unrelated protein, does not stabilize ExoS$^{233\text{-}453}$ during thermal aggregation. (**b**) The PPI inhibitor STO1704 (0.5 mM, final DMSO concentration 2%) counteracts the 14-3-3β induced stabilization of ExoS$^{233\text{-}453}$ during thermal aggregation. Note that the solvent alone also has a destabilizing effect on the complex, likely because it diminishes the polarity around the hydrophobic interface between the two proteins. (**c**) 14-3-3β, at twice molar excess, stabilizes ExoS$^{233\text{-}419}$ during thermal aggregation. All aggregation onset temperatures are given in Supplementary Table 3 below.

Supplementary Table 3: Onset temperatures of heat induced aggregation of ExoS[a]

ExoS construct	Partner or additive	T_{onset} (°C)	Figure
233-453	-	46.7±1.7 (n=3)	4i (main text)
233-453	14-3-3β (0.5:1)[b]	48.0±0.7 (n=3)	4i (main text)
233-453	14-3-3β (1:1)	49.1±1.0 (n=3)	4i (main text)
233-453	14-3-3β (2:1)	50.5±0.4 (n=2)	4i (main text)
233-453	CA (1:1)	49.1 (n=1)	S14a
233-453	CA (2:1)	49.1 (n=1)	S14a
CA[c]	-	60.0 (n=1)	S14a
233-453	14-3-3β (2:1) + 2% DMSO	49.5 (n=1)	S14b
233-453	14-3-3β (2:1) + 0.5mM STO1704	47.2 (n=1)	S14b
233-419	-	36.8 ±1.7 (n=2)	S14c
233-419	14-3-3β (2:1)	49.7 (n=1)	S14c

[a]Thermal aggregation was measured by DLS as described in the Methods section of the main text. The onset temperatures for the thermal transitions are reported. Onset temperatures were determined by interception of the linear fits of the data in the lower temperature range and in the thermal transition. [b]Figures in parentheses indicate molar ratios of additive proteins over ExoS. [c]CA, carbonic anhydrase.

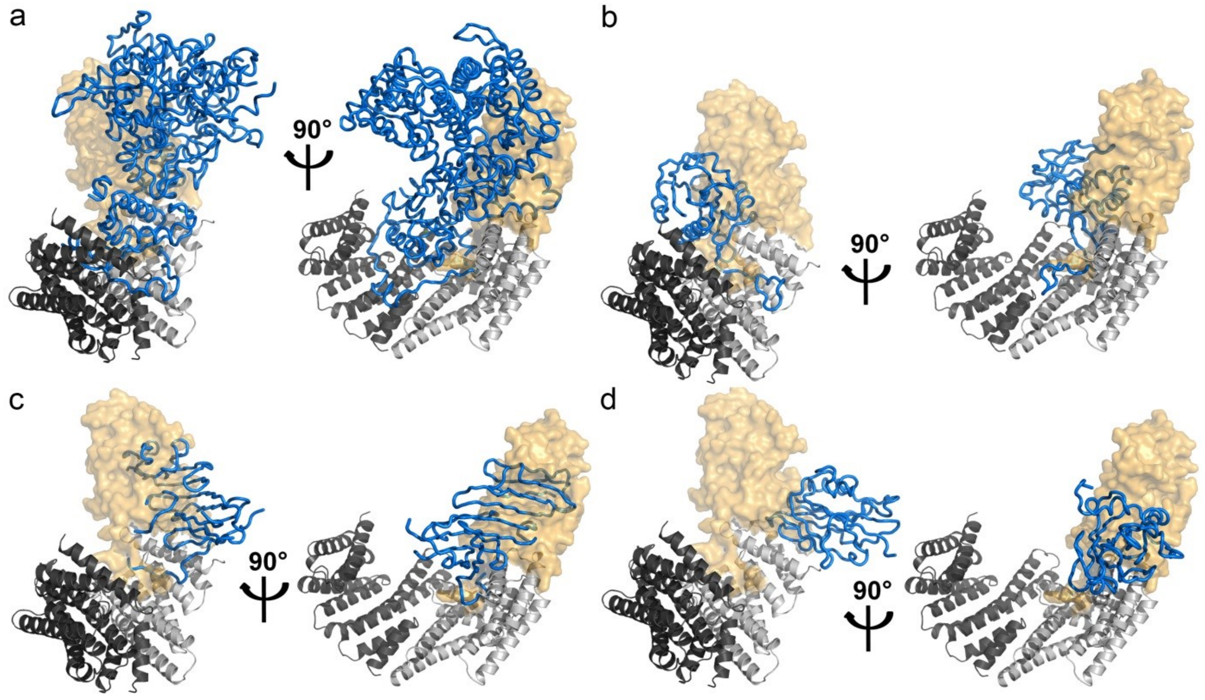

Supplementary Figure 15

Comparison of 14-3-3β:ExoS with four 14-3-3 client complexes. 14-3-3 proteins are shown as cartoons (grey; two shades for the two protomers); ExoS is shown as semi-transparent surface (gold) and other proteins are shown as wireframe (blue). (**a**) Overlay of 14-3-3β:ExoS with the yeast Bmh1:trehalase complex (PDB: 5N6N).[11] The two partner proteins show a significant overlap in their binding sites around the two C-terminal helices of 14-3-3; but the binding mode is different. (**b**) Overlay of 14-3-3β:ExoS with the 14-3-3ζ:serotonin N-acetyltransferase complex (PDB: 1IB1).[12] (**c**) Overlay of 14-3-3β:ExoS with the 14-3-3σ:HspB6 complex (PDB: 5LTW).[13] The contact areas are 1558 Å^2 between 14-3-3 and ExoS and 1063 Å^2 between 14-3-3 and HspB6; 479 Å^2 of these contact areas overlap. (**d**) Overlay of 14-3-3β:ExoS with the 14-3-3 protein GF14:rice florigen Hd3a complex (PDB: 3AXY).[14]

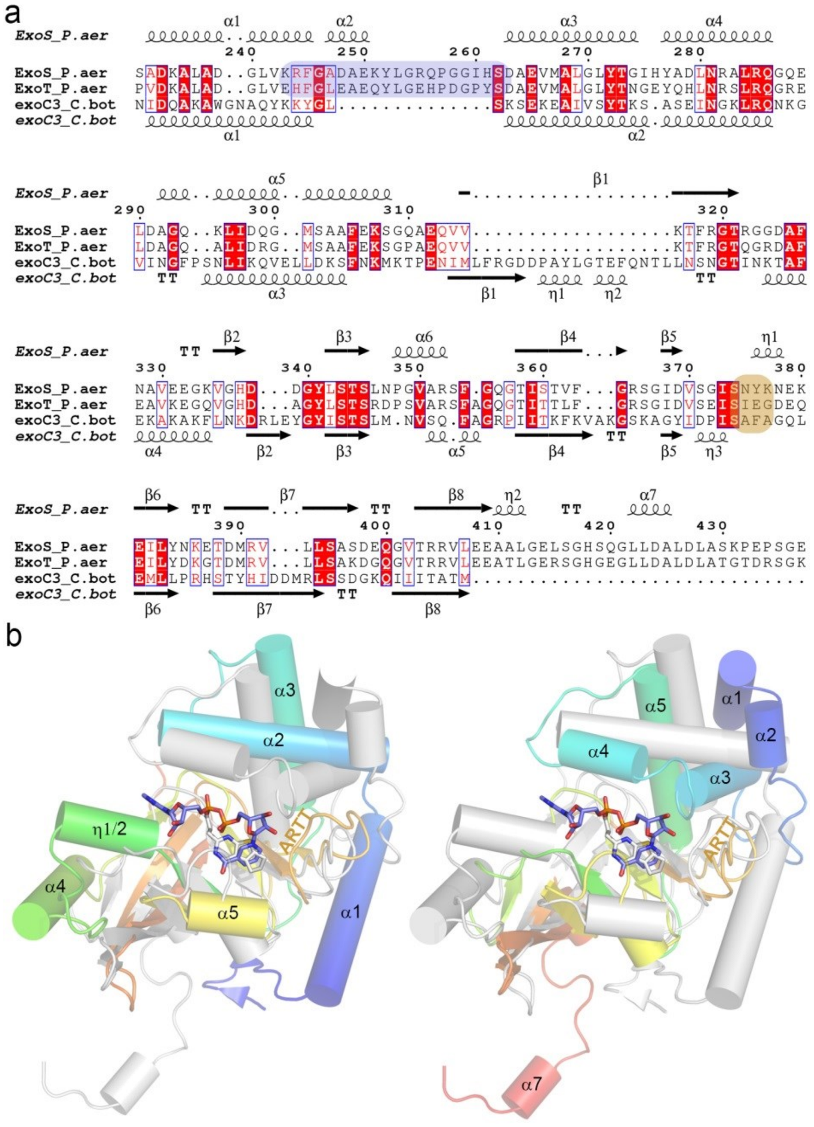

Supplementary Figure 16

Revised positioning of the substrate binding region in ExoS and –T. (**a**) The amino acid sequences of the ExoS, ExoT, and exotoxin C3 ART domains were aligned using default settings in T-Coffee[15] and the secondary structures of ExoS and C3 were added using ESPript.[7] The substrate recognition site of ExoT, identified by Sun and co-workers in 2004,[16] is highlighted in purple and the ARTT loop in pale orange. Despite the generally low overall sequence homology, the homology of key functionalities and their placement in overall topology is readily recognizable. The substrate recognition site of ExoT, on the other hand, falls into a region near the N-terminus of the domain that has an unexpected topology considering the structures of C3 and related toxins. (**b**) Structure alignments of ExoT (PDB: 6GNN) and C3 (PDB: 1GFZ). *Left panel*, ExoT is in grey, with compound STO1101 shown as sticks with carbon atoms in grey. C3 is colored from blue (N-terminus) to red (C-terminus) with bound NAD^+ shown as sticks with carbon atoms in purple. Selected secondary structural elements are named as in panel a. *Right panel*, the same alignment, but ExoT is colored and C3 is in grey. Note the difference in topology in the N-terminal substrate binding regions: Our crystal structures show that unlike those in exotoxin C3, the substrate recognition regions of ExoT and ExoS are in an extended loop adjacent to the ARTT-loop.

Supplementary Table 4: Oligonucleotide primers used in this study

Oligo name	Forward[a]	Reverse[a]
S233_419pNIC	TACTTCCAATCCATGGCCGACAAGGCGCTGGC	TATCCACCTTTACTGTCAGCTATGGCCACTCAGCTC
S233_435pNIC	TACTTCCAATCCATGGCCGACAAGGCGCTGGC	TATCCACCTTTACTGTCAGCTTGCCAGGTCGAGAGC
S419_453Trx	TACTTCCAATCCATGAGCCAGGGACTGCTCGATG	TATCCACCTTTACTGTCAGGCCAGATCGAGGCCAC
S233_419Duet	TTGAAGAATTCGGAATCTTTATTTTCAGGGCGCC	AACATGAAGCTTTCAGCTATGGCCACTCAG
S233_453Duet	GGATCCGAATTCGGAGAATCTTTATTTTCAGGGC	GGCAAGCTTTCAGGCCAGATCGAGGCC
S233_419GFP	TACTTCCAATCCATGGCCGACAAGGCGCTGGC	TATCCACCTTTACTGCTGCTATGGCCACTCAGCTC
S233_435GFP	TACTTCCAATCCATGGCCGACAAGGCGCTGGC	TATCCACCTTTACTGCTGCTTGCCAGGTCGAGAGC
S233_453GFP	TACTTCCAATCCATGGCCGACAAGGCGCTGGC	TATCCACCTTTACTGCTGGCCAGATCGAGGCCAC
S419_453GFP	TACTTCCAATCCATGAGCCAGGGACTGCTCGATG	TATCCACCTTTACTGCTGGCCAGATCGAGGCCAC
T235_457Duet	GGATCCGAATTCTGCCTGTCGACAAGGCGCTG	GGCAAGCTTTCAGGCCAGGTCGAGGCC
1433b_Duet	GGCAGCCATATGACAATGGATAAAAGTGAGCTGG	CCGGTACCTTACTCGAGGGATTGGAAGTACAGG
1433z_pNIC	TACTTCCAATCCATGGATAAAAACGAGCTGGTACAG	TATCCACCTTTACTGTCATCCTGCTTCAGCTTCGTCTC
Rnd1_pNIC	TACTTCCAATCCATGAAGGAGAGACGGGCCCC	TATCCACCTTTACTGTCACATAATGGAACAGCTTTTGGC
Rac3_pNIC	TACTTCCAATCCATGCAGGCCATCAAGTGCGTG	TATCCACCTTTACTGTCAGAAGACGGTGCACTTCTTC

[a] Displayed in the 5' to 3' direction

Supplementary Table 5: Yeast strains used in this study[a]

Name	Genotype
**CB3166 (Vector)**	MATa, ade2-1, trp1-1, can1-100, leu2-3, 112, his3-11, 15, ura3::URA- pGAL1-10 (YIplac211)
**CB3167 (ExoS 233-419)**	MATa, ade2-1, trp1-1, can1-100, leu2-3, 112, his3-11, 15, ura3::URA- pGAL1-10-ExoS$^{233\text{-}419}$ (YIplac211)
**CB3168 (ExoS 233-435)**	MATa, ade2-1, trp1-1, can1-100, leu2-3, 112, his3-11, 15, ura3::URA- pGAL1-10- ExoS $^{233\text{-}435}$ (YIplac211)
**CB3169 (ExoS 233-453)**	MATa, ade2-1, trp1-1, can1-100, leu2-3, 112, his3-11, 15, ura3::URA- pGAL1-10- ExoS $^{233\text{-}453}$ (YIplac211)

[a] Related to **Figure 3f** of the main text.

Supplementary Methods

Crystallization of ExoS:14-3-3β and ExoT:14-3-3β complexes

At the onset of this study, we were unable to produce pure, soluble exotoxins in amounts sufficient for protein crystallization. To identify suitable combinations of exotoxin ART domains and 14-3-3 isoforms, we conducted a comparative analysis of 14-3-3 concentration dependent exotoxin activity *in vitro*. Six of the seven human 14-3-3 isoforms stimulated ExoS enzymatic activity to similar extent; 14-3-3σ had markedly lower apparent affinity; and 14-3-3β was the isoform with the highest apparent affinity for the toxin (**Supplementary Fig. 1**). A 14-3-3β:ExoT^{wt} ART domain co-expression vector was readily obtained; but plasmids encoding 14-3-3β and the ExoS ART domain were apparently highly toxic to *E.coli* cloning strains such that wild type clones could not be obtained. We therefore introduced the active site mutations E379A,E381A[17] in the ExoS cDNA, and obtained clones carrying the resulting co-expression vector. Using immobilized metal ion chromatography we isolated hexahistidine-tagged ADP-ribosyltransferase domains of either toxin (ExoS$^{233\text{-}453(E379A,E381A)}$ or ExoT$^{235\text{-}435(wt)}$) in complex with untagged 14-3-3β.

For both exotoxins ion exchange chromatography on heparin sepharose, as well as consecutive size exclusion chromatography coupled to right-angle light scattering (SEC-RALS), indicated the formation of protein complexes of various constitutions. A protein complex with an apparent molecular weight of roughly 83.5 kDa (**Fig. 1b** of the main text) was indicative of one ExoS^{E379A,E381A} monomer bound to a 14-3-3β dimer ("heterotrimer"; expected molecular weight 83246 Da). This species readily crystallized under various conditions, and buffer screening and optimization resulted in crystals that diffracted to 3.24 Å. The crystals were classified as space group C2 and the asymmetric unit contained one heterotrimer. We solved the structure using molecular replacement with 14-3-3β and Vis toxin (PDB entries 2C23[18] and 4XZJ[6]) as search models (**Supplementary Table 1** and **Fig. 1c** and **Table 1** of the main text). A complex with an apparent molecular weight of roughly 95 kDa ("heterotetramer"; expected molecular weight 104206 Da; **Fig. 1 d** of the main text), yielded crystals of space group $P2_12_12$ that diffracted to 3.24 Å (**Fig. 1e** of the main text). Analysis of the structure showed a crystal packing that allowed the 14-3-3β C-terminus to insert in the active site of ExoS in a neighbor unit cell (**Supplementary Fig. 2**). We re-designed the co-expression vector, substituting the full length 14-3-3β cDNA with a cDNA truncated at N234. Subsequent work resulted in crystal structures of the 14-3-3β:ExoS *apo* heterotrimer at 2.3 Å, the heterotrimer with the non-hydrolyzable NAD analog, carba-NAD in the active site at 2.5 Å, and the heterotrimer with inhibitor STO1101[1] at 3.2 Å resolution (**Fig. 1, Table 1** of the main text). The 14-3-3β:ExoT complexes showed a similar distribution of various molecular weight species (**Supplementary Fig. 1**). We determined the structure of the 14-3-3β:ExoT heterotetramer in complex with STO1101, but the model could not be refined beyond ~3.8 Å resolution.

Carba-NAD synthesis

General methods

^{1}H NMR and ^{13}C NMR spectra were recorded with a Bruker DRX-600 spectrometer. NMR experiments were conducted at 298 K in D_2O (residual solvent peak = 4.79 ppm (δH)). High resolution mass spectra (HRMS) were recorded with an Agilent 1290 binary LC system connected to an Agilent 6230 Accurate-Mass TOF LC/MS (ESI+) that was calibration with the Agilent G1969-85001 ES-TOF Reference Mix containing ammonium trifluoroacetate, purine and hexakis(1H, 1H, 3H tetrafluoropropoxy)phosphazine in 90:10 acetonitrile:water. Organic solvents were dried using a Glass Contour Solvent Systems (SG Water USA) or were dried over molecular sieves 3Å. Flash chromatography was performed on Biotage Isolera One instrument using appropriate SNAP Cartridge KP-Sil Cartridge and UV absorbance at 254 nm. Thin Layer Chromatography (TLC) was performed on Silica gel 60 F254 (Merck). Ion chromatography was performed on a Biotage Isolera One instrument using commercially available aminopropyl-linked silica gel loaded on empty cartridges. Columns were conditioned as previously described.[19]

Chemical syntheses

(1R,4S,5R,6S)-5,6-Dihydroxy-2-azabicyclo[2.2.1]heptan-3-one (2). Potassium osmate dihydrate (50% (w/w), 54 mg, 0.07 mmol) was added to a solution of **1** (800 mg, 7.33 mmol) and N-methylmorpholine N-oxide (945 mg, 8.06 mmol) in isoamyl alcohol/water (1:1 (v/v), 7.4 mL). The reaction was heated at 70 °C for 3 h. After completion, the reaction was quenched by addition of $NaHSO_3$ (382 mg, 3.67 mmol). The mixture was stirred for an additional 45 min while cooling to room temperature. The solvents were then concentrated to dryness under reduced pressure at 40 °C. The solid residue was suspended in a MeOH/iPROH solution (1:1 (v/v), 10 mL) and the mixture was concentrated to dryness. This process was performed twice in order to remove remaining water and N-methylmorpholine. Purification using automated column chromatography (cartridge pre-packed with 50 g of silica gel, 50 mL/min flow rate; 5 min elution at 10% iPrOH in EtOAc, 20 min gradient from 10% to 30% iPrOH in EtOAc, 5 min elution at 30% iPrOH in EtOAc; p-Anisaldehyde was used as TLC-stain) afforded **2** as a white solid (75% yield). ^{1}H NMR and ^{13}C NMR data were in agreement with literature.[19]

^{1}H NMR (600 MHz, D_2O) δ 4.08 (d, 1H, J = 5.9 Hz), 4.04 (d, 1H, J = 5.9 Hz), 3.82-3.78 (m, 1H), 2.66-2.62 (m, 1H), 2.11-2.07 (m, 2H); ^{13}C NMR (150 MHz, D_2O) δ 182.0, 71.7, 68.3, 59.3, 51.8, 36.2; HRMS (ESI) *m/z* ($[M+H]^+$) calcd for $C_6H_{10}NO_3$ 144.0661, found 144.0652.

(1S,2R,3S,4R)-Methyl 4-Amino-2,3-dihydroxycyclopentane- carboxylate Hydrochloride (3). Compound **2** (800 mg, 5.59 mmol) was dissolved in MeOH (10 mL) (note: gentle heating was needed to fully solubilize the starting material). HCl(g) was bubbled into the reaction mixture for 3 h and the mixture was stirred overnight at room temperature. Next, the solution was

concentrated to dryness under reduced pressure to give **3** as a white crystalline solid (quant.; 83% purity). ^{1}H NMR and ^{13}C NMR data were in agreement with literature.[19]

^{1}H NMR (600 MHz, D_2O) δ 4.31 (t, 1H, J = 5.3 Hz), 4.09 (dd, 1H, J = 7.2, 5.4 Hz), 3.76 (s, 3H), 3.59 (q, 1H, J = 8.3 Hz), 3.02 (dt, 1H, J = 9.0, 5.0 Hz), 2.54 (dt, 1H, J = 13.8, 8.6 Hz), 1.87 (dt, 1H, J = 13.8, 9.2 Hz); ^{13}C NMR (150 MHz, D_2O) δ 175.36, 74.17, 72.60, 54.41, 52.73, 47.53, 27.13; HRMS (ESI) *m/z* ($[M+H]^+$) calcd for $C_7H_{14}NO_4$ 176.0922, found 176.0907.

(1R,2S,3R,5R)-3-Amino-5-(hydroxymethyl)cyclopentane-1,2- diol (4). Dry THF (30 mL) was added to compound **3** (500 mg, 2.36 mmol) and the mixture was stirred at 0 °C under nitrogen atmosphere. Lithium triethylborohydride (1M in THF, 11.81 mmol) was slowly added to the reaction and the suspension became clear as the addition was proceeding. Next, the reaction mixture was stirred for an additional 30 min at 0 °C. The solvent was then removed under reduced pressure. The solid residue was dissolved in MeOH and the mixture was acidified with aqueous HCl (0.1 M). After removal of the solvents, the crude product was dissolved in water (5 mL) and loaded on a column containing Dowex® 50WX8 (H^+ form). The column was washed with water until neutral pH. Then, the compound was eluted using aqueous ammonia (1 M). The product containing fractions were concentrated under reduced pressure until full removal of ammonia. The remaining aqueous solution was freeze dried to yield **4** as a yellow oil (quant., 90% purity). ^{1}H NMR and ^{13}C NMR data were in agreement with literature.[19]

^{1}H NMR (600 MHz, D2O) δ 3.87 (t, 1H, J = 5.0 Hz), 3.65-3.55 (m, 3H), 3.21 (dt, 1H, J = 8.9, 7.6 Hz), 2.24-2.15 (m, 1H), 2.12-2.03 (m, 1H), 1.05 (dt, 1H, J = 13.0, 9.1 Hz); ^{13}C NMR (150 MHz, D_2O) δ 78.74, 73.38, 64.04, 55.33, 45.21, 31.24; HRMS (ESI) *m/z* ($[M+H]^+$) calcd for $C_6H_{14}NO_3$ 148.0974, found 148.0968.

3-Carbamoyl-1-((1R,2S,3R,4R)-2,3-dihydroxy-4-(hydroxymethyl)cyclopentyl)pyridin-1-ium Chloride (6). A solution of **5**[19] (220 mg, 0.68 mmol) in MeOH (0.5 mL) was added to a solution of **4** (141.5 mg, 0.71 mmol) in MeOH (2.5 mL) at room temperature. The reaction turned purple upon addition. Then, sodium acetate (55.6 mg, 0.68 mmol) was added and the reaction mixture was stirred at room temperature for 4 h. Aqueous ammonia (17 M, 0.15 mL) was added and the reaction was stirred for an additional 10 min. The solvents were removed under reduced pressure and the residue was suspended in water. The precipitate was filtered off and the filtrate was concentrated under reduced pressure. MeOH was added to the resulting oil and the solvents were removed under reduced pressure. The crude product was taken up in MeOH and directly loaded on a 25 g silica gel pre-packed cartridge. Purification was performed as follow: 25 mL/min flow rate; 15 min gradient from 5% to 100% MeOH (containing 5% (v/v) AcOH) in EtOAc, then 5 min elution with 100% MeOH (containing 5% (v/v) AcOH). The product containing fractions were concentrated to dryness, dissolved in water and acidified with HCl(conc.). The solvents were then concentrated to dryness to give **6** as a yellow hygroscopic solid (73% yield). ^{1}H NMR and ^{13}C NMR data were in agreement with literature.[19]

^{1}H NMR (600 MHz, D_2O) δ 9.42 (s, 1H), 9.17 (d, 1H, J = 6.1 Hz), 8.97 (d, 1H, J = 8.1 Hz), 8.27 (dd, 1H, J = 7.8, 6.2 Hz), 5.11 (ddd, 1H, J = 10.4, 9.4, 8.0 Hz), 4.46 (dd, 1H, J = 9.4, 6.1 Hz), 4.13 (dd, 1H, J = 5.8, 3.1 Hz), 3.77 (d, 2H, J = 5.9 Hz), 2.70 (dt, 1H, J = 13.4, 8.3 Hz), 2.41-2.32 (m, 1H), 2.01 (ddd, 1H, J = 13.1, 11.1, 8.9 Hz); ^{13}C NMR (150 MHz, D_2O) δ 166.45, 146.04, 145.43, 143.77, 134.80, 129.26, 77.35, 76.23, 72.20, 63.26, 45.32, 29.90; HRMS (ESI) *m/z* ([M+H]$^+$) calcd for $C_{12}H_{17}N_2O_4$ 253.1188, found 253.1178.

((1R,2R,3S,4R)-4-(3-Carbamoylpyridin-1-ium-1-yl)-2,3- dihydroxycyclopentyl)methyl Hydrogen Phosphate (7). Prior to reaction start, **6** (hygroscopic compound, 125 mg, 0.43 mmol) was dissolved in MeOH in the reaction vessel and concentrated to dryness under reduced pressure. This process was performed twice and, then, the reaction vessel was placed under vacuum for 1 h. Next, the vessel was backfilled with nitrogen and fresh trimethyl phosphate (1.1 mL) was added. The resulting suspension was stirred for 1 min under high vacuum at room temperature, backfilled with nitrogen and cooled at 0 °C. $POCl_3$ (121 µL, 1.30 mmol) was then added via a degassed syringe. The starting material dissolved within a few minutes after addition and the reaction was stirred at 0 °C for 1.5 h. The reaction was quenched by addition of water (0.15 mL) and then stirred for 40 min at 0 °C. Next, sodium acetate (533 mg, 6.49 mmol) in MeOH (32.5 mL) was added at room temperature. The compound was purified using a cartridge containing 10 g of propyl amino functionalized silica previously equilibrated with 0.1 M acetic acid in methanol. The crude product in MeOH was loaded on the column at a flow rate of 10 mL/min. 50 mL of acetic acid 0.1 M in methanol were eluted to remove all reagents and MeOH soluble compounds. 50 mL AcOH 0.1 M in water were then eluted to collect the compound. The compound containing fractions were concentrated to dryness under reduced pressure at 40 °C. The oil was diluted in water and concentrated under reduced pressure to remove most of the acetic acid traces. This process was done twice. The oil was then dissolved in water and freeze dried to yield **7** as a yellow solid (78% yield). ^{1}H NMR and ^{13}C NMR data were in agreement with literature.[19]

^{1}H NMR (600 MHz, D_2O) δ 9.42 (t, 1H, J = 1.4 Hz), 9.20 (dd, 1H, J = 6.3, 1.3 Hz), 8.96 (dd, 1H, J = 8.2, 1.3 Hz), 8.27 (dd, 1H, J = 8.0, 6.3 Hz), 5.12 (td, 1H, J = 9.8, 8.8 Hz), 4.52 (dd, 1H, J = 9.6, 5.7 Hz), 4.21 (dd, 1H, J = 5.7, 2.5 Hz), 4.10-4.04 (m, 1H), 4.01-3.95 (m, 1H), 2.77 (dt, 1H, J = 13.6, 8.8 Hz), 2.52-2.44 (m, 1H), 2.16 (ddd, 1H, J = 13.6, 10.6, 7.9 Hz); ^{13}C NMR (150 MHz, D_2O) δ 166.36, 145.94, 145.53, 143.74, 134.68, 129.27, 77.70, 76.44, 72.87, 66.49 (d, J_{C-P} = 5.4 Hz), 43.77 (d, J_{C-P} = 8.2 Hz), 29.55; HRMS (ESI) *m/z* ([M+H]$^+$) calcd for $C_{12}H_{18}N_2O_7P$ 333.0852, found 333.0844.

1-((1R,2S,3R,4R)-4-(((((((2R,3S,4R,5R)-5-(6-Amino-9H-purin-9-yl)-3,4-dihydroxytetrahydrofuran-2-yl)-methoxy)hydroxyphosphoryl)oxy)oxidophosphoryl)oxy)-methyl)-2,3-dihydroxycyclopentyl)-3-carbamoylpyridin-1-ium (Carba-NAD, 8). Pyridine (0.28 mL) and formamide (1.4 mL) were added to a mixture of **7** (91 mg, 0.27 mmol), adenosine 5-monophosphomorpholidate 4-morpholine-N,N-dicylco-hexylcarboxamidine salt

(389 mg, 0.55 mmol), p-toluenesulfonic acid monohydrate (141 mg, 0.74 mmol) and anhydrous $MnCl_2$ (103 mg, 0.82 mmol). The reaction was stirred at room temperature under nitrogen atmosphere for 1 day. The reaction mixture was placed in a syringe, and the reaction vessel was washed with 6 drops of water. The washings were added to the syringe containing the reaction mixture, and subsequently loaded on the equilibrated column loaded with 10 g of propyl amino functionalized silica. The column was eluted at a flow rate of 30 mL/min with (1) 150 mL of 0.1 M acetic acid in MeOH (to recover the morpholinecarboxamidine salt, formamide, and pyridine as a single peak), (2) 182 mL of 0.1 M acetic acid and 0.1 M sodium acetate in MeOH (to recover sodium p-toluenesulfonate), (3) 91 mL of 0.1 M acetic acid in MeOH (to remove sodium acetate), (4) 121 mL of 0.1 M aqueous acetic acid (to recover unreacted **7** and some $MnCl_2$), (5) 121 mL of 0.01 M aqueous acetic acid (to decrease the concentration of acetic acid on the column), and (6) 121 mL of 0.15 M sodium acetate/0.01 M acetic acid in water (to elute Carba-NAD). The column was washed with 1 M sodium acetate and 1 M acetic acid in water to remove the rest of the reaction byproducts. The column was then equilibrated with 0.1 M acetic acid in MeOH. The Carba-NAD containing fractions were concentrated under reduced pressure at 40 °C until sodium acetate began to crystallize (residual volume about 2-4 mL). The residue was dissolved in 7 mL of MeOH, and then 0.7 mL of 0.5 M EDTA in water (pH = 8) was added to sequester traces of Mn^{2+}. The solution was concentrated under reduced pressure to remove as much of the water as possible, and then the residue was taken up in 7 mL of MeOH and injected on the amino functionalized silica gel column, equilibrated with 0.1 M acetic acid in MeOH. The column was eluted at a flow rate of 30 mL/min with 300 mL of 0.1 M acetic acid in MeOH (to recover sodium acetate) and then with 300 mL of 1 M acetic acid in water (to recover Carba-NAD). The Carba-NAD containing fractions were concentrated under reduced pressure at 40 °C to a thick, colorless oil. This was taken up in water and concentrated under reduced pressure (3 × 5 mL) to remove the remaining acetic acid. The oily residue was then taken up in 5 mL of water and freeze dried to give Carba-NAD (**8**) as a colorless, amorphous solid (48% yield). ^{1}H NMR and ^{13}C NMR data were in agreement with literature.[19]

^{1}H NMR (600 MHz, D_2O) δ 9.40 (t, 1H, J = 1.3 Hz), 9.18 (dt, 1H, J = 6.3, 1.2 Hz), 8.91 (dt, 1H, J = 8.2, 1.4 Hz), 8.62 (s, 1H), 8.41 (s, 1H), 8.23 (dd, 1H, J = 8.1, 6.3 Hz), 6.16 (d, 1H, J = 5.6 Hz), 5.09 (q, 1H, J = 10.0 Hz), 4.78 (t, 1H, J = 5.3 Hz), 4.50-4.56 (m, 2H), 4.39-4.43 (m, 1H), 4.31−4.11 (m, 3H), 4.20-4.14 (m, 1H), 4.11-4.05 (m, 1H), 2.72 (dt, 1H, J = 13.6, 8.8 Hz), 2.50-2.43 (m, 1H), 2.22 (ddd, 1H, J = 13.8, 10.9, 7.9 Hz); ^{13}C NMR (150 MHz, D_2O) δ 166.15, 151.80, 149.21, 147.11, 146.27, 145.69, 143.56, 142.58, 134.63, 129.33, 119.23, 88.36, 84.90 (d, J_{C-P} = 8.9 Hz), 77.82, 76.64, 75.22, 73.02, 71.00, 67.42 (d, J_{C-P} = 5.6 Hz), 65.86 (d, J_{C-P} = 5.6 Hz), 43.77 (d, J_{C-P} = 8.6 Hz), 29.52; HRMS (ESI) *m/z* ($[M+H]^+$) calcd for $C_{22}H_{30}N_7O_{13}P_2$ 662.1377, found 662.1356.

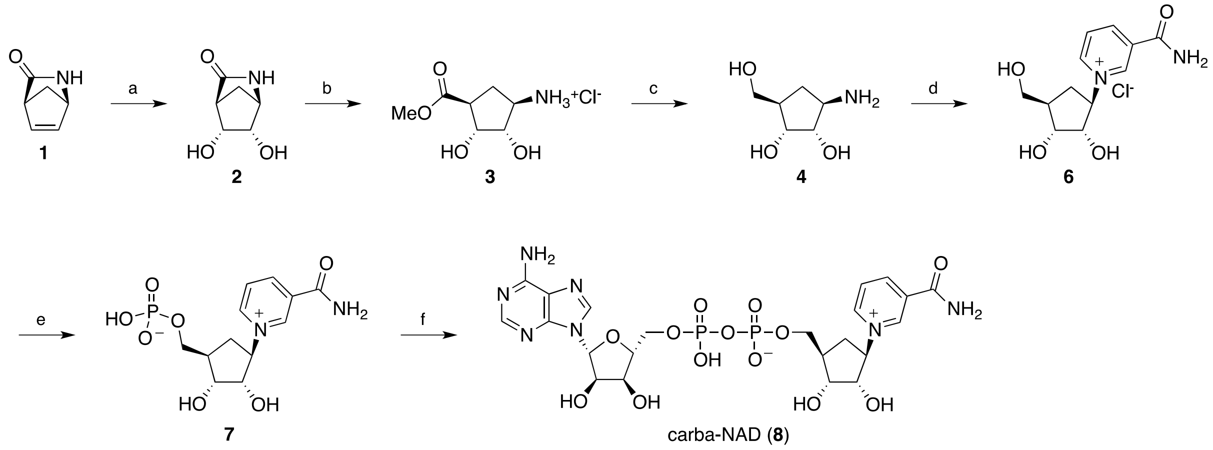

Supplementary Figure 17

Chemical synthesis of carba-NAD. Reagents and conditions: (a) $K_2OsO_4.2H_2O$, NMO, H_2O/Isoamyl alcohol, 60 °C (75%); (b) HCl(g), MeOH, rt, (quant.); (c) i. $LiEt_3BH$, THF, 0 °C, ii. HCl(aq.), MeOH (quant.); (d) 3-carbamoyl-1-(2,4-dinitrophenyl)pyridin-1-ium chloride (**5**), NaOAc, MeOH, rt (73%); (e) $POCl_3$, trimethyl phosphate, 0 °C (78%); (f) $MnCl_2$, pyridine, p-TsOH, adenosine 5-monophosphomorpholidate 4-morpholine-N,N-dicylcohexylcarboxamidine salt, formamide (48%).

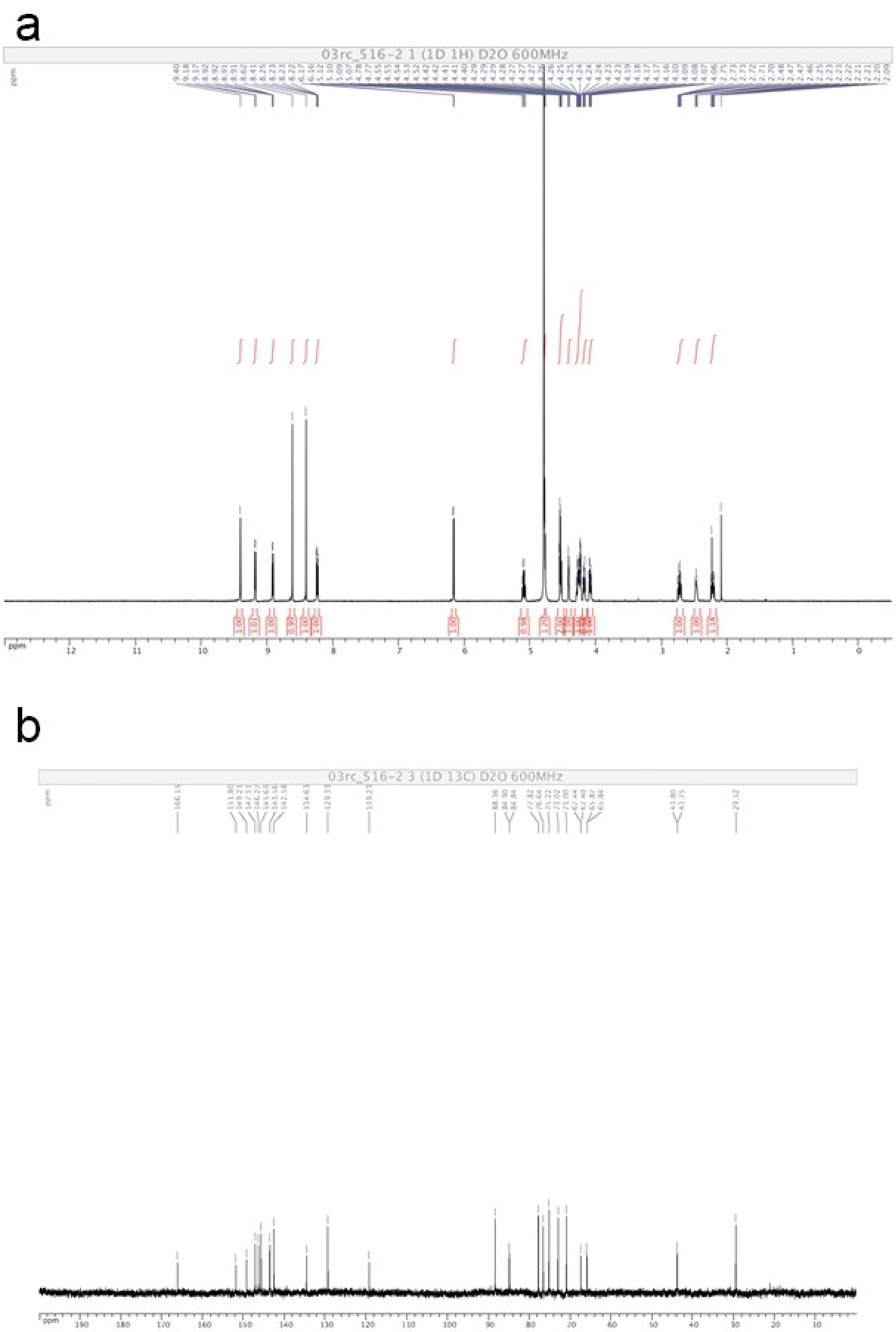

Supplementary Figure 18

NMR spectra for carba-NAD. (**a**) ^{1}H NMR spectrum of carba-NAD in D_2O, recorded at 600 MHz. (**b**) ^{13}C NMR spectrum of carba-NAD in D_2O, recorded at 150 MHz.

Supplementary References

1. Pinto, A.F. et al. Identification of Inhibitors of Pseudomonas aeruginosa Exotoxin-S ADP-Ribosyltransferase Activity. *J. Biomol. Screen.* **21**, 590-595 (2016).
2. Saleeb, M. et al. Structure-activity relationships for inhibitors of Pseudomonas aeruginosa exoenzyme S ADP-ribosyltransferase activity. *Eur. J. Med. Chem.* **143**, 568-576 (2017).
3. Laskowski, R.A. & Swindells, M.B. LigPlot+: multiple ligand-protein interaction diagrams for drug discovery. *J. Chem. Inf. Mod.* **51**, 2778-2786 (2011).
4. Krissinel, E. Stock-based detection of protein oligomeric states in jsPISA. *Nucl. Acid Res.* **43**, W314-319 (2015).
5. Li, W. et al. The EMBL-EBI bioinformatics web and programmatic tools framework. *Nucl. Acid Res.* **43**, W580-584 (2015).
6. Ravulapalli, R. et al. Characterization of Vis Toxin, a Novel ADP-Ribosyltransferase from Vibrio splendidus. *Biochemistry* **54**, 5920-5936 (2015).
7. Gouet, P., Courcelle, E., Stuart, D.I. & Metoz, F. ESPript: analysis of multiple sequence alignments in PostScript. *Bioinformatics* **15**, 305-308 (1999).
8. Sijbesma, E. et al. Identification of Two Secondary Ligand Binding Sites in 14-3-3 Proteins Using Fragment Screening. *Biochemistry* **56**, 3972-3982 (2017).
9. Cromm, P.M. et al. Constraining an Irregular Peptide Secondary Structure through Ring-Closing Alkyne Metathesis. *ChemBioChem* **17**, 1915-1919 (2016).
10. Henriksson, M.L. et al. Exoenzyme S shows selective ADP-ribosylation and GTPase-activating protein (GAP) activities towards small GTPases in vivo. *Biochemical J.* **367**, 617-628 (2002).
11. Alblova, M. et al. Molecular basis of the 14-3-3 protein-dependent activation of yeast neutral trehalase Nth1. *Proc. Natl. Acad. Sci. U. S. A.* **114**, E9811-E9820 (2017).
12. Obsil, T., Ghirlando, R., Klein, D.C., Ganguly, S. & Dyda, F. Crystal structure of the 14-3-3zeta:serotonin N-acetyltransferase complex. a role for scaffolding in enzyme regulation. *Cell* **105**, 257-267 (2001).
13. Sluchanko, N.N. et al. Structural Basis for the Interaction of a Human Small Heat Shock Protein with the 14-3-3 Universal Signaling Regulator. *Structure* **25**, 305-316 (2017).
14. Taoka, K. et al. 14-3-3 proteins act as intracellular receptors for rice Hd3a florigen. *Nature* **476**, 332-335 (2011).
15. Notredame, C., Higgins, D.G. & Heringa, J. T-Coffee: A novel method for fast and accurate multiple sequence alignment. *J. Mol. Biol.* **302**, 205-217 (2000).

16. Sun, J., Maresso, A.W., Kim, J.J. & Barbieri, J.T. How bacterial ADP-ribosylating toxins recognize substrates. *Nat. Struct. Mol. Biol.* **11**, 868-876 (2004).
17. Radke, J., Pederson, K.J. & Barbieri, J.T. Pseudomonas aeruginosa exoenzyme S is a biglutamic acid ADP-ribosyltransferase. *Infect. Immun.* **67**, 1508-1510 (1999).
18. Yang, X. et al. Structural basis for protein-protein interactions in the 14-3-3 protein family. *Proc. Natl. Acad. Sci. U. S. A.* **103**, 17237-17242 (2006).
19. Szczepankiewicz, B.G. et al. Synthesis of carba-NAD and the structures of its ternary complexes with SIRT3 and SIRT5. *J. Org. Chem.* **77**, 7319-7329 (2012).
